# Integration of microRNA signatures of distinct mammary epithelial cell types with their gene expression and epigenetic portraits

**DOI:** 10.1186/s13058-015-0585-0

**Published:** 2015-06-18

**Authors:** Bhupinder Pal, Yunshun Chen, Andrew Bert, Yifang Hu, Julie M. Sheridan, Tamara Beck, Wei Shi, Keith Satterley, Paul Jamieson, Gregory J. Goodall, Geoffrey J. Lindeman, Gordon K. Smyth, Jane E. Visvader

**Affiliations:** ACRF Stem Cells and Cancer Division, The Walter and Eliza Hall Institute of Medical Research, Parkville, VIC 3052 Australia; Department of Medical Biology, The University of Melbourne, Parkville, VIC 3010 Australia; Bioinformatics Division, The Walter and Eliza Hall Institute of Medical Research, Parkville, VIC 3052 Australia; Centre for Cancer Biology, SA Pathology and University of South Australia, Adelaide, SA 5000 Australia; Department of Computing and Information Systems, The University of Melbourne, Parkville, VIC 3010 Australia; School of Medicine and School of Molecular and Biomedical Science, University of Adelaide, Adelaide, SA 5005 Australia; Department of Medicine, The University of Melbourne, Parkville, VIC 3010 Australia; Department of Medical Oncology, The Royal Melbourne Hospital, Parkville, VIC 3010 Australia; Department of Mathematics and Statistics, The University of Melbourne, Parkville, VIC 3010 Australia

## Abstract

**Introduction:**

MicroRNAs (miRNAs) have been implicated in governing lineage specification and differentiation in multiple organs; however, little is known about their specific roles in mammopoiesis. We have determined the global miRNA expression profiles of functionally distinct epithelial subpopulations in mouse and human mammary tissue, and compared these to their cognate transcriptomes and epigenomes. Finally, the human miRNA signatures were used to interrogate the different subtypes of breast cancer, with a view to determining miRNA networks deregulated during oncogenesis.

**Methods:**

RNA from sorted mouse and human mammary cell subpopulations was subjected to miRNA expression analysis using the TaqMan MicroRNA Array. Differentially expressed (DE) miRNAs were correlated with gene expression and histone methylation profiles. Analysis of miRNA signatures of the intrinsic subtypes of breast cancer in The Cancer Genome Atlas (TCGA) database versus those of normal human epithelial subpopulations was performed.

**Results:**

Unique miRNA signatures characterized each subset (mammary stem cell (MaSC)/basal, luminal progenitor, mature luminal, stromal), with a high degree of conservation across species. Comparison of miRNA and transcriptome profiles for the epithelial subtypes revealed an inverse relationship and pinpointed key developmental genes. Interestingly, expression of the primate-specific miRNA cluster (19q13.4) was found to be restricted to the MaSC/basal subset. Comparative analysis of miRNA signatures with H3 lysine modification maps of the different epithelial subsets revealed a tight correlation between active or repressive marks for the top DE miRNAs, including derepression of miRNAs in *Ezh2-*deficient cellular subsets. Interrogation of TCGA-identified miRNA profiles with the miRNA signatures of different human subsets revealed specific relationships.

**Conclusions:**

The derivation of global miRNA expression profiles for the different mammary subpopulations provides a comprehensive resource for understanding the interplay between miRNA networks and target gene expression. These data have highlighted lineage-specific miRNAs and potential miRNA–mRNA networks, some of which are disrupted in neoplasia. Furthermore, our findings suggest that key developmental miRNAs are regulated by global changes in histone modification, thus linking the mammary epigenome with genome-wide changes in the expression of genes and miRNAs. Comparative miRNA signature analyses between normal breast epithelial cells and breast tumors confirmed an important linkage between luminal progenitor cells and basal-like tumors.

**Electronic supplementary material:**

The online version of this article (doi:10.1186/s13058-015-0585-0) contains supplementary material, which is available to authorized users.

## Introduction

The ductal epithelial networks that characterize mouse and human mammary tissue appear to comprise an analogous cellular hierarchy: multi-potent mammary stem cells (MaSCs) reside at the apex of the hierarchy and are capable of differentiation along the myoepithelial/basal lineage or the luminal lineage to yield mature ductal and alveolar cells [1–6]. The precise nature of the intermediate cell types remains unclear but two or three distinct luminal progenitor subsets have been prospectively isolated from mouse and human mammary tissue, respectively [4, 7–9]. Several functional studies have used a candidate approach to identify regulators of self-renewal, lineage commitment and differentiation programs (reviewed in [10]). Furthermore, genome-wide transcriptome analyses [11, 12] of mouse mammary epithelial subsets have identified a number of potential regulators of mammary gland development. The definition of numerous conserved pathways across species has highlighted those that are likely to be involved in cell-fate decisions and lineage differentiation [12]. Moreover, the epigenome has been implicated in playing a critical role in regulating such decisions within the epithelial compartment of the normal mammary gland [13, 14].

There is increasing evidence that microRNAs (miRNAs) regulate a wide range of biological processes, including maintenance of cell identity, differentiation and apoptosis [15–17]. miRNAs, small non-coding RNA molecules that inhibit translation or trigger mRNA decay [15, 17], have been implicated in both mammary gland development and breast tumorigenesis. In a large-scale study, the expression of 318 miRNAs was assessed during different stages of development, leading to the observation that miRNAs can be expressed in coordinated clusters, and that global miRNA and mRNA expression are significantly lower in lactation and early involution [18]. In the mouse mammary epithelial cell line, Comma-Dβ [19], the expression of miR-205 and miR-22 but not let-7 and miR-93 was linked to progenitor-like properties, while miR-200c appears to function within the basal cell compartment of normal breast tissue [20]. Interestingly, miR-200c targets the mRNA encoding BMI1, a key regulator of the self-renewal of stem cells in multiple tissues. MiR-193b also has been implicated in regulating mammary stem cell activity in vivo and may serve an additional function in controlling the alveolar differentiation during pregnancy [21]. In the context of breast cancer, many miRNAs have been reported to undergo deregulation, inferring an important role in controlling proliferation versus differentiation decisions. For example, miR-205 is one the most significantly downregulated miRNAs in human breast cancer relative to normal tissue [22]. Moreover, miRNA signatures that distinguish breast tumors of different subtypes from normal tissue have been described [23]. To understand the consequences of deregulated miRNA networks, it is essential to characterize the normal expression patterns and roles of miRNAs in the epithelial differentiation hierarchy. Here we sought to determine the global miRNA expression profiles of discrete cellular subpopulations within normal mouse and human mammary tissue. Comparative analyses of miRNA signatures with gene expression and histone modification profiles of the epithelial subsets revealed candidate miRNAs that are likely to execute important roles in mammary epithelial specification and differentiation.

## Methods

### RNA preparation and quantitative PCR analysis

The cellular subsets isolated by flow cytometry have been previously described [12]. Mice were on a pure FVB/N background. All experiments were approved by the Animal Ethics Committee of the Walter and Eliza Hall Institute of Medical Research, and the care of animals was in accordance with institutional guidelines. Experiments using human tissue obtained from the Victorian Cancer Biobank, each with patient consent, were approved by the Human Research Ethics Committees of The Walter and Eliza Hall Institute of Medical Research and Melbourne Health.

Total RNA or miRNA populations were isolated from primary mammary cell subpopulations using the miRNeasy kit (Qiagen, Germany). Notably, identical cell pellets (following sorting) were used for mRNA and miRNA preparation: the transcriptomes for these subsets are reported in Lim et al. [12]. Reverse transcription was carried out using oligo(dT) primer and SuperscriptIII reverse transcriptase (Invitrogen, MA, USA). For miRNA samples, reverse transcription was performed using a target-specific stem loop primer and reverse transcriptase (Invitrogen) according to the manufacturer’s protocol. Quantitative RT-PCR was carried out using a Rotorgene RG-6000 (Corbett Research, Australia) under the following conditions: 2 minutes at 50 °C and 10 minutes at 95 °C followed by 35 cycles consisting of 15 s at 95 °C and 60 s at 60 °C. Gene expression was determined using Rotor-Gene software (version 1.7).

### microRNA expression profiling

At least three biological replicates of RNA from each human and mouse cell subpopulation were profiled for miRNA expression using a Taqman array system (Applied Biosystems, MA, USA). Complementary DNA (cDNA) was prepared using TaqMan miRNA Reverse Transcription Kit (ABI 4366596) and the Megaplex RT primer pools (mouse v2.0 ABI #4401012 and human v2.0 ABI #4401091) according to the manufacturer’s protocol. For each sample, cDNA was made using appropriate Megaplex primer pools and 3 μl RNA as a template. Each pool was pre-amplified for 12 cycles using TaqMan PreAmp Master Mix (ABI #4391128) and Megaplex preAmp primers (mouse v2.0 ABI #4401012 and human v2.0 ABI #4401091). Quantitative RT-PCR was performed on an ABI 7900HT machine using either the TaqMan Rodent MicroRNA Array Set v2.0 (ABI #4400239) or Taqman Human MicroRNA Set v2.0 (ABI #4400238), Taqman Low Density Array plates and Universal PCR Master Mix (ABI #4364341) according to the manufacturer’s protocol. U6 was included as an endogenous control and Arabidopsis thaliana-miR159a was included as a negative control on each plate. Ct (cycle threshold) values were exported using SDS v2.3 and RQ Manager v1.2 software (Applied Biosystems, MA, USA), with automatic baseline and a manual Ct threshold of 0.2.

### Statistical analyses of microRNA expression values in normal cell subpopulations

The maximum measurable Ct value was 40, so Ct values were transformed to a log2 expression scale by subtracting the Ct values from 40.5. Statistical analysis was carried out using the limma software package [24]. The expression values were normalized using cyclic loess normalization [25] with house-keeping probes up-weighted 100-fold. The cyclic method was set to “affy”, the loess span was 0.7, and five cyclic iterations were used. For mouse, the snRNA U6 was the house-keeping probe. For human, U6, RNU6B, RNU24, RNU43, RNU44 and RNU48 were all treated as house-keeping probes. For both mouse and human, probes were filtered out as unexpressed if they failed to achieve a normalized value of 2 in at least three samples. The RT-PCR expression data is available from the Gene Expression Omnibus as superseries GSE67056.

Comparisons were made between cell populations using empirical Bayes t- and F-statistics [26]. The false discovery rate (FDR) was controlled below 0.05 using the method of Benjamini and Hochberg [27]. For each subpopulation (MaSC/basal, luminal progenitor, and mature luminal), signature probes were defined as those that were differentially expressed (DE) versus the average of the other two cell subpopulations. Separate analyses were conducted for mouse and human. A combined analysis was also conducted using the mouse and human data together, using a linear model that included a covariate to adjust for differences between species. For the combined analysis, mouse and human Taqman probes were matched by miRNA symbols.

### Correlation of microRNAs and putative target mRNAs

TargetScan [28] was used to identify putative target mRNAs for each miRNA. ROAST gene set tests [29] were applied to test whether the expression level of each miRNA was negatively correlated with the expression of its target mRNAs. The ROAST tests were conducted using mRNA expression values obtained from Illumina BeadChips as previously published [12]: GEO series GSE19446 for mouse data and GSE16997 for human data. A one-sided *P*-value was obtained for each miRNA to test whether the average log-fold expression change of the target genes was in the opposite direction to that of the miRNA.

Gene ontology (GO) analysis was conducted using the goana function of the limma package. Genes were selected as DE for the purposes of GO analysis if they were (i) a target of a DE miRNA and (ii) DE in the mRNA microarray data with FDR <0.2 in the inverse direction to the miRNA.

### Analysis of microRNA signatures in breast cancer subtypes

miRNA profiles of breast cancer tumors were downloaded from The Cancer Genome Atlas (TCGA) Data Portal [30], specifically from the data directory bcgsc.ca_BRCA.IlluminaHiSeq_miRNASeq.Level_3.1.17.0. Only profiles of primary solid tumors were used for this analysis. PAM50 tumor subtype calls were obtained for each sample from the TCGA analysis working party (CM Perou and KA Hoadley, personal communication). The data consist of Illumina HiSeq read counts for each miRNA in each sample. Samples with the same analyte IDs were treated as technical replicates and were combined by summing their read counts for each miRNA. Similarly, counts for different isoforms of the same miRNA were summed. miRNAs were filtered as unexpressed if they failed to achieve at least one read per million in at least 29 samples. This left data on 451 miRNAs for 720 tumor samples. Using the edgeR package, counts were normalized by the trimmed mean of M-values method [31] and then converted to log2 counts per million with a prior count of 0.25.

For each tumor sample, miRNA expression signature scores were computed to measure similarity with MaSC-enriched, luminal progenitor and mature luminal cells, using a method similar to that used previously for mRNA expression scores [2]. Given a set of signature genes for each cell subset and associated log2 fold changes, expression scores were computed as sum(logFC * logCPM)/sums(abs(logFC)), where logFC is the log2 fold change for a miRNA between normal cell subsets in the PCR data and logCPM is the log2 count per million for the miRNA from the RNA-seq data. The sum is taken over all miRNAs. An expression score was computed for each tumor and epithelial cell subset.

### Analysis of epigenetic modifications of microRNA loci

Genome-wide ChIP-seq profiles of H3K4me3 and H3K27me3 histone marks were obtained previously for the mouse MaSC/basal, luminal progenitor and mature luminal cell subsets [14]. Sequence data is available from the Gene Expression Omnibus series GSE43212. For this study, we re-aligned the reads to the mouse genome mm10 using the subread aligner [32]. Genomic locations of miRNAs were downloaded from miRBase [33]. Read counts were obtained for a genomic interval extending 3 kb upstream and 3 kb downstream from the body of each miRNA using featureCounts [34]. The same genomic interval was used for both 3′ and 5′ isoforms of the same miRNA where these existed. A differential binding analysis between the three epithelial subsets was undertaken using the edgeR Bioconductor package [35]. Log2 fold changes in binding intensity between the three epithelial subsets were computed for each miRNA using edgeR’s glmFit function, setting the negative binomial dispersion to 0.05 and the prior count to 1 [36]. The prior count has the effect of shrinking the log2 fold changes slightly towards zero to avoid unstable fold changes for low counts. The ChIP-seq log2 fold changes were correlated with miRNA expression log2 fold changes using regression through the origin.

## Results

### Lineage-specific expression of mammary microRNAs

Freshly sorted cellular subsets corresponding to MaSC/basal, luminal progenitor, mature luminal and stroma cells were prospectively isolated from mouse and human mammary glands. In the mouse, the immunophenotypes of the subpopulations [3] are: CD29^hi^CD24^+^ (MaSC/basal), CD29^lo^CD24^+^CD61^+^ (luminal progenitor), CD29^lo^CD24^+^CD61^–^ (mature luminal) and CD29^–^CD24^–^ (stroma). For human breast, the phenotypes [2] are: CD49f^hi^EpCAM^–/lo^ (MaSC/basal), CD49f^+^EpCAM^+^ (luminal progenitor), CD49f^lo^EpCAM^+^ (mature luminal) and CD49f^–^EpCAM^–^ (stroma). Of note, the MaSC/basal population comprises stem cells, putative basal progenitor cells and mature myoepithelial cells. The miRNA studies were performed on the same sorted human and mouse cellular subsets as used previously for mRNA expression profiling using microarrays [12]. As for the transcriptome study, a minimum of three biological replicate samples were profiled from each cell subpopulation: cDNA was prepared for miRNA profiling from the small RNA fraction (less than 200 bp), and mouse and human Megaplex RT Primer Pools containing primers for either 585 mouse or 667 human miRNAs (plus species-specific controls) were utilized. A pre-amplification step (12 cycles) was incorporated to improve the chance of detecting miRNAs expressed at very low levels. High throughput RT-PCR was used to generate log2 expression values for all miRNAs and samples.

**Fig. 1:**
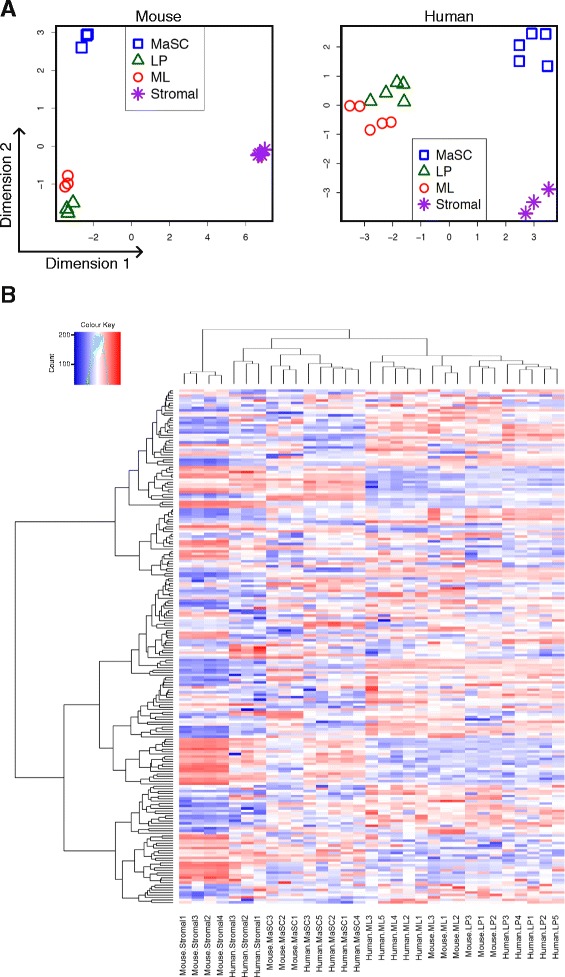
Global miRNA expression in sorted populations from mouse and human mammary glands. Mouse and human epithelial subsets are marked by unique miRNA expression signatures. **a** Multi-dimensional scaling plots indicate clear separation of the mammary stem cell (MaSC)/basal-enriched, luminal progenitor (LP), mature luminal (ML) and stromal subsets in both mouse (*left panel*) and human (*right panel*). Distances on the plot represent the log2 fold change for a typical miRNA between the samples. Stromal cells are well separated from the epithelial subsets, with a typical expression change of around 5 logs or 32-fold. The MaSC/basal subset is well separated from the luminal subsets with a typical expression change of around 4 logs or 16-fold. **b** Combined heat map shows hierarchical clustering of all conserved miRNAs in mouse and human epithelial subsets including stromal subsets (*red* = high expression; *blue* = low expression)

Each cellular subpopulation was marked by a distinct miRNA expression pattern (Fig. 1). In both mouse and human, stromal cells were well separated from the three epithelial subpopulations (Fig. 1a). Differential expression analysis comparing the stroma subset with the average of the three epithelial subsets revealed 276 miRNAs that distinguished stroma from the total epithelium in mouse (Additional file 1: Table S1; FDR <0.05) and 181 in human (Additional file 2: Table S2; FDR <0.05).

The three epithelial subpopulations were also distinct. Analysis of variance found 221 miRNAs that were DE between the MaSC/basal, luminal progenitor and mature luminal populations in mouse (Additional file 3: Table S3, FDR <0.05) and 209 in human (Additional file 4: Table S4, FDR <0.05). The greatest expression differences were associated with the MaSC/basal subsets. The progenitor and mature luminal subpopulations showed relatively closer expression profiles while still being distinct from each other. Comparison of the MaSC/basal subset with the average of the two luminal populations revealed 188 differentially expressed miRNAs in mouse. Of these, 107 miRNAs were more highly expressed in the mouse MaSC/basal subset and 81 were more highly expressed upon restriction to the luminal lineage (Additional file 5: Table S5; FDR <0.05). The same comparison in human found 213 differentially expressed miRNAs between the MaSC/basal subset and the luminal lineage, with 163 upregulated in the MaSC/basal subset and 50 in luminal cells (Additional file 6: Table S6; FDR <0.05).

### Conservation across species

To explore the mouse and human data together, a batch correction was used to adjust for differences between the two species and hierarchical clustering was applied to all the mouse and human cell populations together (Fig. 1b). This analysis was restricted to miRNA families found in both species. The clustering confirmed a clear separation between the stromal, MaSC/basal and luminal cell populations, with all cell subsets clustering together despite species differences (Fig. 1b). In particular, the mouse and human stromal populations clustered together despite known differences between stroma in the two species. Mouse mammary stroma is known to comprise a higher proportion of adipocytes, whereas human breast stroma is highly enriched for fibroblasts. The homologous expression profiles between human and mouse stroma suggest that either population might be utilized to support human breast epithelial cells in cell-based assays ex vivo.

A differential expression analysis of the combined mouse and human expression profiles was conducted to find miRNAs that showed the same pattern of differential expression between the epithelial subsets in both species. Analysis of variance revealed 111 miRNAs that were consistently differentially expressed between the three epithelial subsets (FDR <0.05). A more focused comparison of the MaSC/basal subset with the combined luminal subsets found 108 differentially expressed miRNAs, of which 50 had higher expression in the MaSC/basal subset and 58 in the luminal subsets (Additional file 7: Table S7, FDR <0.05). Top conserved miRNAs in the MaSC/basal population include miR-204 (may target *ERα*), miR-221/222 (targets *ERα* and *c-Kit*) [37, 38], and miR-205 (targets *Pten* and *Bcl-2*) [39, 40]. Luminal-restricted miRNAs included miR-10a (targets *KLF4* and *PIK3CA*) [41, 42], miR-200a/b (targets EMT (epithelial mesenchymal transition) genes) [43], miR-148a (targets *Bim*) [44] and miR-375 (targets *PDK1*) [45].

### Target gene prediction and inverse correlation between microRNA and mRNA expression in distinct subpopulations

**Fig. 2:**
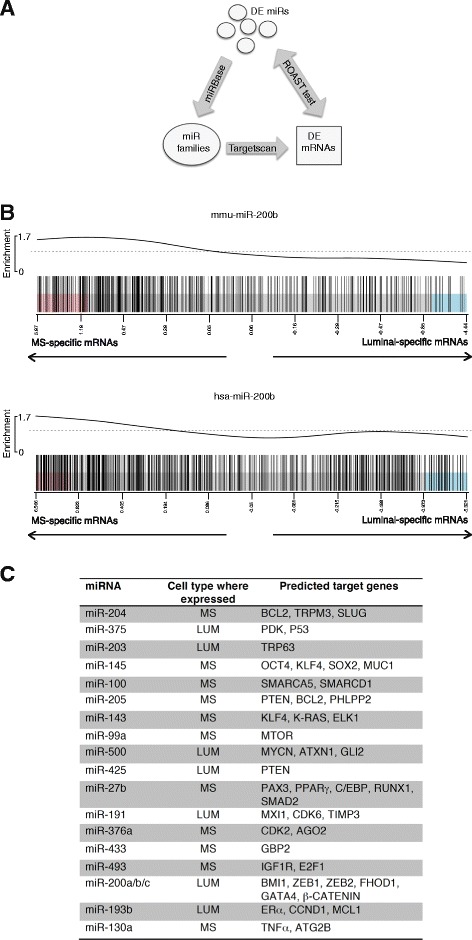
Inverse correlation between differentially expressed miRNAs in specific subpopulations and their transcriptomes. Lineage-specific miRNAs are conserved between mouse and human mammary tissue. **a** Schematic representation of Rotation Gene Set Test (ROAST) analysis [29]. Mouse and human Taqman probes were matched by miRNA symbols obtained from the miRNA database (miRBase) and TargetScan was used to relate miRNAs to target mRNAs. ROAST tests were performed to detect miRNAs that are most negatively correlated with their target mRNAs. **b** Barcode plots showing the expression patterns of the mouse and human mRNA targets of the conserved luminal-specific miR-200b. Genes are ranked in terms of relative expression from highest in MaSC/basal cells (MS) to highest in luminal cells. Target genes are marked with vertical bars and the worm shows relative enrichment. The target genes tend to be less highly expressed in the luminal than the MaSC/basal subset. **c** Predicted target genes for the top 20 conserved differentially expressed miRNAs in the two major populations. *DE* differentially expressed, *miRNA miR* microRNA, *Lum* combined luminal progenitor and mature luminal cells

To explore the potential biological functions of DE miRNAs between mammary epithelial lineages, we identified putative target mRNAs for each miRNA. This was achieved using the Targetscan program, which predicts miRNA-binding sites in mRNA 3′ untranslated regions. Many of the miRNAs specific to the MaSC/basal subset (Additional file 3: Table S3 and Additional file 4: Table S4) were observed to target key luminal-lineage mRNAs including *Gata3, Notch1, Elf5, c-Kit and Esr1* [11, 12]*.* Conversely, many luminal-specific miRNAs have been implicated in targeting transcription factors that are restricted to basal cells in the mammary gland such as *Snai2* and *Trp63* [11, 12]. Predicted target mRNAs for a number of miRNAs are shown in Fig. 2c. Many of these are likely to be relevant to lineage restriction in the mammary gland such as miR-203, which is expressed in luminal cells and targets the basal-restricted genes *Snai2* and *Trp63* [46–48].

To rigorously test whether the DE miRNAs are in fact regulating their putative mRNA target genes within a given mammary lineage, we carried out Rotation Gene Set Tests (ROAST) to assess whether the expression of each miRNA was inverse correlated with that of its target genes during luminal commitment. The expression levels of mRNA genes in the mouse and human MaSC/basal, luminal progenitor and mature luminal subpopulations were measured by microarrays as previously reported [12]. This analysis confirmed that many of the DE miRNAs between the MaSC/basal and luminal subsets do show significant inverse correlations with their target mRNAs, supporting the hypothesis that they constitute an active regulatory mechanism (Additional file 8: Table S8). This was true for both mouse and human. As a representative example, the inverse correlation is displayed by barcode plots for the mouse and human versions of miR-200b (Fig. 2b), which is a key miRNA that targets the EMT genes *Zeb1 and Zeb2* [43].

GO enrichment analysis was used to examine the biological processes and molecular functions that are regulated as basal stem/progenitor cells commit to the luminal lineage. In particular, GO analysis was conducted on the putative mRNA targets of miRNAs that were differentially expressed between the MaSC/basal and luminal subsets. This revealed that luminal-specific miRNAs tend to downregulate signaling pathways including cell differentiation, cell development, and regulation of developmental process in both mouse and human mammary epithelium (Additional file 9: Table S9A and Additional file 10: Table S10A), whereas MaSC/basal-specific miRNAs tend to downregulate processes characteristic of differentiated cells including intracellular localization, transport, organelle, biosynthesis, secretion and cell–cell interaction pathways (Additional file 9: Table S9B and Additional file 10: Table S10B).

### Restricted expression of a primate-specific microRNA cluster in the MaSC/basal subset

**Fig. 3:**
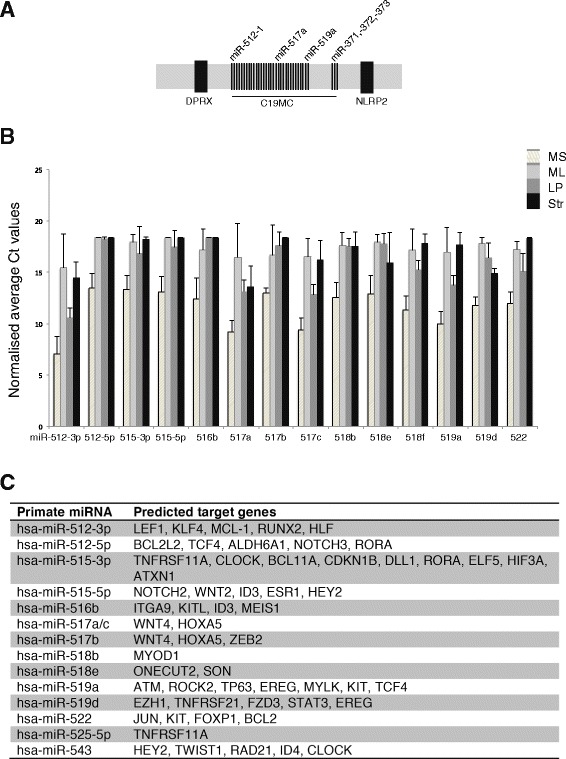
The human MaSC/basal population is enriched for primate-specific miRNAs. Primate-specific miRNAs are expressed at higher levels in the human MaSC/basal subset (MS) relative to luminal and stromal cells. **a** The primate-specific chromosome-19 miRNA cluster (C19MC) is located at 19q13.4, flanked by the DPRX and NLRP2 genes. C19MC is ~150 kb in length and codes for 50 known miRNAs. **b** Bar plots show normalized average Ct values for 15 primate-specific miRNAs that are differentially expressed between the MaSC/basal (MS), mature luminal (ML), luminal progenitor (LP) and stromal (str) cell subsets. Quantitative PCR data was normalized against the U6 small RNA (n = 3 independent biological samples; error bars represent SEM). **c** The table shows known or putative mRNA targets of DE primate-specific miRNAs in human breast epithelium

Analysis of human miRNA profiles revealed differential expression of primate-specific miRNAs between the basal and luminal epithelial subsets. Interestingly, the region localized to chromosome 19q13.4 harbors a miRNA cluster that spans ~150 kb and encodes 50 miRNAs (C19MC, Fig. 3a). The expression of these miRNAs has been reported to be high during embryonic development and in human embryonic stem cells [49, 50]. Significant expression of miRNAs within this cluster (miR-512-3p, miR-512-5p, mir-515-5p, miR-516b, miR-517a, miR-517c, miR-518b, miR-518f, miR-519a and miR-519d) was observed in the MaSC/basal population (Fig. 3b), whereas no highly expressed luminal-specific primate miRNAs were identified. Targetscan analysis indicated *RANK* (TNFRSF11A), a member of the TNF superfamily of receptors, and *MCL-1*, a BCL-2 pro-survival family member, as potential target genes. These observations are compatible with the very low expression of *Rank* and *Mcl-1* mRNA in the MaSC/basal subset [12, 51]. Moreover, the luminal lineage-restricted genes, *ERα* and *ELF5*, were predicted to be targeted by DE primate-specific miRNAs (Fig. 3c). The other major primate-specific miRNA cluster miR-506-514 localized on chromosome X [52, 53] did not show differential expression amongst the human breast epithelial subsets.

### Comparison of microRNA signatures for normal human breast epithelial subpopulations versus breast cancer subtypes

**Fig. 4:**
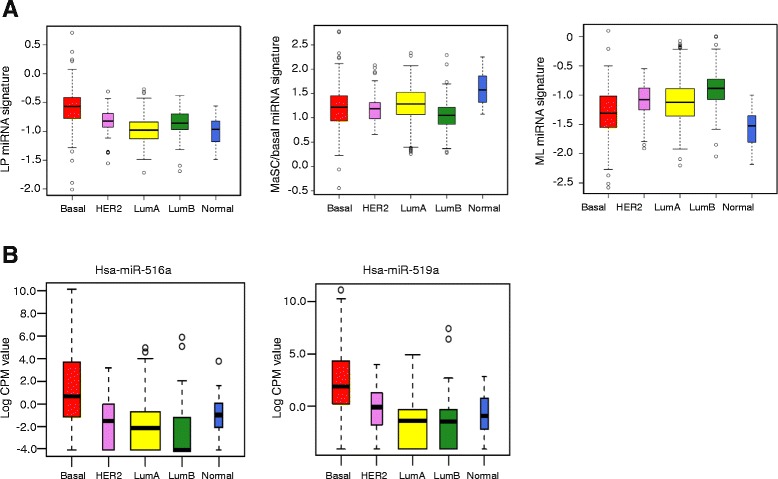
Comparison of normal human breast epithelial miRNA signatures with miRNA profiles of different breast cancer subtypes. **a** The miRNA signatures of human mammary epithelial subsets were used to identify relationships between normal human epithelial subsets and tumors of different molecular subtypes. The miRNA signature scores of epithelial subsets were derived as MS vs LP+ML; LP vs MS+ML and ML vs MS+LP (*MS* MaSC/basal, *LP* luminal progenitor, *ML* mature luminal). Panels show boxplots for the LP, MS and ML signature expression scores with respect to the miRNA signatures of tumor subtypes derived from the TCGA database (breast cancer subtype: *LumA* luminal A, *LumB* luminal B, *HER2* Human epidermal growth factor receptor 2 positive, *Basal* often triple negative tumors). **b** Expression of basal-restricted, primate-specific miRNAs was examined in the different subtypes of breast cancer using the TCGA dataset. Boxplots are shown for miRNA-516a and miR-519a, where significant enrichment was observed in the basal-like subtype. *CPM*, counts per million

Breast cancer has been stratified into at least five molecular subtypes [54] and miRNA profiling of 101 primary breast tumors revealed differential expression of miRNAs between these different subgroups [55]. Moreover, large-scale miRNA profiling of breast cancers carried out under the TCGA project has provided a comprehensive list of differentially expressed miRNAs between the different subtypes [56]. Here we utilized miRNA signatures to identify potential relationships between normal human epithelial subsets and tumors of different molecular subtypes, analogous to that performed using mRNA signatures [2]. The MaSC/basal miRNA signature was found to be highest in the normal-like subtype of breast cancer (*P* < 1.6 × 10^–6^), while the mature luminal signature was closest to the luminal B subtype (*P* < 9.4 × 10^–5^) (Fig. 4a). Interestingly, the luminal progenitor miRNA signature was highest in the basal-like subtype relative to all other subtypes (*P* < 1.4 × 10^–9^), reminiscent of that observed for their transcriptomes [2]. Interrogation of the TCGA database for expression of DE primate-specific miRNAs (listed in Fig. 3c) revealed considerable enrichment of miR-516a and miR-519a in basal-like tumors (Fig. 4b; *P* < 1.9 × 10^–5^ and *P* < 5.3 × 10^–9^, respectively), in contrast to the other miRNAs, which did not demonstrate enrichment (data not shown).

### Correlation of microRNA profiles with epigenetic modification

**Fig. 5:**
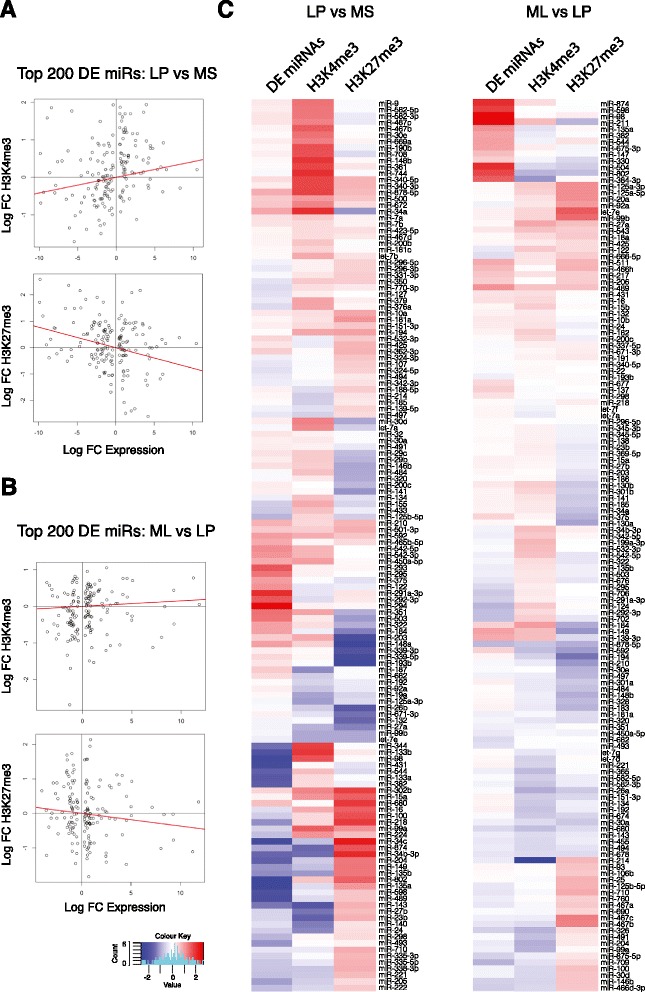
Correlation between miRNA expression and histone methylation patterns. The histone marks H3K4me3 and H3K27me3 correlate with miRNA expression. Results are shown for the top 200 (scatter plot) or 140 (heatmap) differentially expressed miRNAs between the luminal progenitor (*LP*) and MaSC/basal (*MS*) subsets and for the top 200 (scatter plot) or 140 (heatmap) differentially expressed (*DE*) miRNAs between the mature luminal (*ML*) and LP subsets. **a** Scatter plots show that expression changes between the MS and LP subsets are directly correlated with differential H3K4me3 marking (*top panel*, *P* = 0.017) and inversely correlated with H3K27me3 marking (*bottom panel*, *P* = 9.6 × 10^-5^). **b** Scatter plots show that expression changes between the ML and LP subsets are uncorrelated with differential H3K4me3 marking (*top panel*, *P* = 0.4) but inversely correlated with H3K27me3 marking (*bottom panel*, *P* = 0.108). **c** Heatmaps of expression and epigenetic changes. Vertical columns represent log2-fold expression changes for expression, H3K4me3 binding and H3K27me3 binding, respectively. The left panel clusters the same log-fold changes as for (**a**). The right panel clusters the same log-fold changes as for (**b**) (*red* = increased; *blue* = decreased). *FC* fold change

**Fig. 6:**
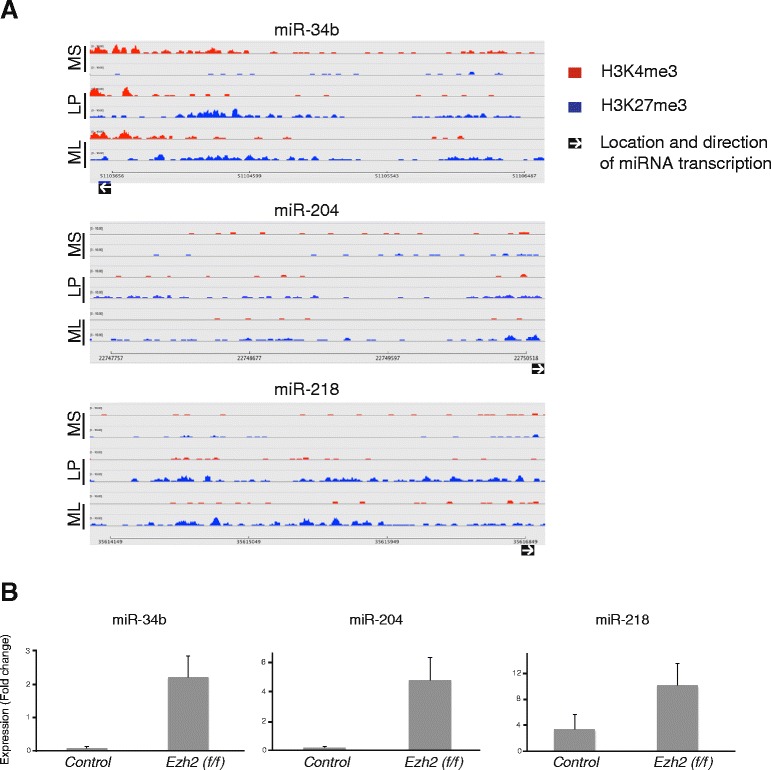
Ezh2 mediates repression of mammary epithelial miRNAs. MiR-34b, miR-204 and miR-218 are expressed highly in the MaSC/basal subset. **a** Track files or read coverage graphs for H3K4me3 and H3K27me3 marks present in the 3 kb upstream region of miR-34b (*top panel*), miR-204 (*middle panel*) and miR-218 (*bottom panel*) in each epithelial subset. Y-axes show fragments per million on the scale 0–10. **b** Bar graphs represent quantitative RT-PCR analysis for mammary epithelial cells isolated from control (Ezh2^f/+^) or Ezh2-deficient (MMTV-cre;Ezh2^f/f^) mammary glands. Expression of the MaSC/basal-specific miRNAs miR-34b, miR-204 and miR-218 is upregulated in luminal cells of *Ezh2-*deficient samples compared to littermate controls. Data was normalized against U6 small RNA. n = 3 independent biological samples; error bars show SEM. *LP* luminal progenitor, *ML* mature luminal, *MS* MaSC/basal

We recently established the genome-wide histone methylation profiles for the MaSC/basal, luminal progenitor and mature luminal epithelial subsets isolated from the mouse mammary gland and showed that they varied dramatically amongst the two primary lineages and in response to ovarian hormones [14]. To determine whether the expression of miRNAs in the mammary gland was subject to epigenetic regulation, we utilized ChIP-seq data for H3K4me3 and H3K27me3 (activation and repressive marks, respectively) to investigate histone methylation of the regulatory regions of miRNAs spanning a region 3 kb upstream of their putative transcriptional start-site (TSS). Scatter plots (Fig. 5a) of DE genes between the MaSC/basal and luminal progenitor populations demonstrated a positive correlation between miRNA expression and H3K4me3 chromatin modifications, in which the upstream regions of the top 200 DE miRNAs were examined. Conversely, H3K27me3 marks negatively correlated with the top DE miRNAs between these subsets. Interestingly, similar correlations could be seen upon interrogation of luminal progenitor versus mature luminal cells (Fig. 5b), specifically for H3K27 trimethylation. The heat map depicts the relative levels of H3K4me3 and H3K27me3 modifications across a 3 kb upstream region for the top 140 DE miRNAs (Fig. 5c) in the MaSC/basal versus luminal progenitor subsets and the luminal progenitor versus mature luminal subsets. These represent miRNAs and genes that are potentially involved in cell-fate decisions and luminal differentiation, respectively. Representative track file histograms for the MaSC/basal-specific miRs-34b/c, miR-204 and miR-218, highlighting the distribution of H3K27me3 and H3K4me3 peaks, are shown in Fig. 6a. Overall, these data indicate that epigenetic modifications play an important role in lineage restriction along the mammary differentiation hierarchy.

Since Ezh2 is a core enzymatic subunit of the polycomb repressor complex that catalyzes K27 trimethylation on H3, we investigated the role of Ezh2-mediated repression of miRNAs. MiRNAs were extracted from MaSC/basal and luminal cells sorted from either control or Ezh2-deficient mouse mammary glands, and quantitative RT-PCR was then performed for the MaSC/basal-specific miRNAs miR-34b, miR-204 and miR-218, as their promoter regions were enriched for H3K27me3 marks in the luminal subsets (Fig. 6a). In Ezh2-deficient glands, expression of these miRNAs was derepressed in luminal cells, owing to loss of H3K27me3 (Fig. 6b). Conversely, we observed derepression of luminal-specific miR-34a, miR-205 and miR-222 in the MaSC/basal subset of Ezh2-deficient glands (data not shown). Together these findings indicate that Ezh2 plays an important role in the methylation of H3K27 on the promoter regions of miRNAs.

## Discussion

This study describes genome-wide miRNA expression profiling of four distinct mouse and human subpopulations that are highly enriched for MaSC/basal, luminal progenitor, mature luminal and stromal cells. The four subpopulations exhibited distinct miRNA signatures that were conserved across species. Evaluation of potential target genes revealed that the top differentially expressed miRNAs likely target lineage-specific mRNAs. The predicted miRNA–mRNA relationships were found to be highly conserved between mouse and human epithelial subtypes. Around 58 % (25 miRNAs) of the top negatively correlated miRNAs in the mouse mammary gland (Additional file 8: Table S8) were conserved in human; these miRNAs are likely to govern important mammary cell fate and differentiation decisions during ontogeny. The top negatively correlated (conserved) mouse miRNAs include miR-30a/d (targets *Runx2*) [57], miR-148a (targets *Met/Snail*) [58], miR-503 (targets *Bcl-2* and *Igf1r*, implicated in involution) [59], miR-203 (targets the transcription factor *p63*) [60] and miR-34a (targets *Dll1* and *CD44*, important for stem cell activity) [61, 62].

There is accumulating evidence that the Wnt and Notch pathways, as well as the Polycomb repressor complex of proteins, play prominent roles in regulating MaSC function [14, 63–65]. In the context of miRNAs that potentially control these pathways, we identified several luminal-restricted miRNAs, including miR-10a, miR-200a/b, miR-203, miR-148a. Conversely, miR-146a, miR-221/222, and miR-205, which have been shown to regulate genes expressed in the ductal and alveolar luminal lineages (e.g., *Brca1, Gata3, c-kit* and *Elf5)*, were restricted to the MaSC/basal population.

Intriguingly, the primate-specific miRNA cluster (C19MC miRNAs) on chromosome 19 at q13.4 was highly expressed in MaSC/basal cells. Moreover, miR-512 has been implicated in targeting the pro-survival gene *MCL-1* that is expressed at very low levels in this subset [66]. Our Targetscan analysis further identified the luminal-specific genes *RANK, NOTCH3, ELF5, ESR1, HEY2* and *KIT* as potential targets of these primate-specific miRNAs (Fig. 3c). Notably, structural rearrangements of the chromosomal 19q13 region that occur in some thyroid adenomas and adenomatous goiters are associated with aberrant expression of miRNAs in this cluster. In addition, miR-517c and miR-591a are highly expressed in the basal-like subtype of breast cancer [55], further implicating C19MC miRNAs in carcinogenesis.

It has been presumed that the expression of miRNAs and their host genes largely coincide. However, the expression of miRNAs located within introns or the coding regions of specific genes may be independent of host gene expression and its epigenetic modifications. For example, expression of the *BTG4* gene, which harbors the MaSC/basal-specific miRNAs miR-34b and miR-34c, and the *TRP3* gene that encompasses miR-204, is not detectable in mammary epithelium. In addition, the *MIB-1* gene, which is host to the MaSC/basal-specific miRNAs miR-1 and miR-133a, is expressed at very low levels in all three epithelial subsets (data not shown).

The epigenetic landscape of mammary epithelial cells appears to play an important role in the progressive commitment of MaSC/basal cells to differentiated cells. Not only does the epigenome contribute to gene expression changes [14], but it tightly correlates with the miRNA expression profiles of the different mouse mammary epithelial subsets. Despite a paucity of information on the TSS of miRNAs, a clear pattern has emerged for histone methylation marks on DE miRNAs: the top DE miRNAs repressed upon restriction of MaSC/basal cells to luminal progenitor cells were enriched for H3K27me3 modifications, while those activated upon commitment were characterized by the presence of H3K4me3 marks. Similar epigenetic patterning held for the luminal progenitor versus mature luminal populations. Overall, the presence of H3K4me3 marks correlated tightly with the expression of both miRNAs and mRNAs, while H3K27me3 modifications negatively correlated with their expression. Moreover, the histone methylase Ezh2 was directly implicated in coordinating H3K27 trimethylation of the regulatory regions of miRNAs whose expression was repressed. Collectively, these data suggest that miRNA expression is regulated by epigenetic modifications and contributes to decisions on proliferation versus differentiation in the mammary gland. It remains to be determined whether steroid hormones also influence the epigenome of regions flanking miRNA loci. In human mammary epithelial cell lines, the expression of the miR-200 family was recently found to be subject to epigenetic regulation, whereby DNA methylation and histone modifications were altered during the transition between stem-like and nonstem states [67]. DNA methylation of the miR-200c-141 cluster and polycomb group-mediated histone methylation of the miR-200b-200a-429 cluster resulted in repression at these loci [67]. Moreover, H3K4me3 was found to be associated with active miRNAs in colorectal cancer cell lines, whereas hypermethylation of promoter CpG islands caused epigenetic silencing of miR-124 and mir-34b/c [68–71].

Comparison of miRNA signatures derived for distinct mammary epithelial subsets from normal mammary tissue with those of different breast cancer subtypes further strengthened the molecular links that have been previously defined at the mRNA level. Specifically, the miRNA signature of the luminal progenitor population was most concordant with the basal-like cancer subtype, the mature luminal cell-enriched population was closest to the luminal B subtype, and the signature of the MaSC/basal population was highest in the normal-like subtype of cancer. These findings suggest that defined cell types in normal breast tissue may be predisposed to acquiring oncogenic events that result in specific types of cancer. Notably, there was a strong correlation between the miRNA signatures of the luminal progenitor cell and the basal-like subtype of cancer, also reflected in their corresponding transcriptomes. This cell is the likely ‘cell of origin’ for basal-like cancers that arise in BRCA1 mutation carriers [2].

Several highly expressed miRNAs have been associated with the development and progression of breast cancer, in which their aberrant expression is presumed to destabilize mRNAs encoding crucial tumor suppressors and differentiation-promoting factors [72, 73]. Profiling studies of primary breast tumors have revealed differential miRNA expression according to estrogen receptor (ER)/progesterone receptor (PR) or human epidermal growth factor receptor 2 (HER2) status and different tumor stages [23, 55, 74, 75]. More specifically, the expression of some miRNAs has been linked to histopathological features such as HER2/*neu* or ER/PR status (miR-30), metastasis (miR-126 and miR-335) and the EMT (miR-205 and miR-200 family) [43, 76–79]. The luminal subtypes of breast cancer appear to have elevated expression of miR-190b, while basal-like tumors have higher levels of miR-18a/b, miR-9 and the miR-17-92 family and lower levels of miR-29 and miR-190b [55]. The higher levels of miR-18a/b, miR-9 and miR-17-92 in the MaSC/basal population suggest that a subset of triple negative cancers may harbor an expression signature that more closely resembles that of the stem cell population. Furthermore, the primate-specific, basal-restricted miR-516a and miR-519a were most highly expressed in this subtype of breast cancer (Fig. 4b). Other miRNAs recently implicated in breast cancer include miR-100, shown to target SMARCA5, SMARCD1, and BMPR2 genes, which directly influence tumor cell proliferation [80], and miR-30c, known to target TWF1 and IL-11 [81], both of which are expressed in the MaSC/basal lineage. Ultimately, a comprehensive analysis of miRNAs deregulated in breast cancer, together with an understanding of their transcriptional and epigenetic control, may provide novel prognostic or therapeutic tools for breast cancer.

## Conclusions

These global miRNA profiles provide a valuable resource for functional exploration of the molecular and epigenetic regulation of the mammary epithelial hierarchy. Here, our analysis of distinct human and mouse epithelial subtypes has highlighted potential miRNA networks responsible for governing lineage commitment and differentiation in mammary tissue. They further point to relationships between the signatures of normal cell types and intrinsic breast cancer subtypes, supporting the notion that the cell of origin may be an important determinant of tumor pathology. These relationships could be exploited to identify improved biomarkers and small molecule inhibitors of oncogenic pathways.

## Additional files

Additional file 1: Table S1. miRNAs that are differentially expressed between mouse mammary stroma and total epithelium (MS + LP + ML). Differential expression was assessed using a moderated *t*-test for each miRNA. The Table shows log2 fold changes for stroma versus epithelium. All miRNAs with FDR <0.05 are shown. Additional file 2: Table S2. miRNAs that are differentially expressed between human mammary stroma and total epithelium (MS + LP + ML). Differential expression was assessed using a moderated *t*-test for each miRNA. The Table shows log2 fold changes for stroma versus epithelium. All miRNAs with FDR <0.05 are shown. Additional file 3: Table S3. miRNAs that are differentially expressed between the mouse mammary epithelial subsets: MaSC/basal (MS), luminal progenitor (LP) and mature luminal (ML). Differential expression was assessed using a moderated analysis of variance (ANOVA) F-test for each miRNA between the three populations. The Table shows log2 fold changes for LP versus MS and for ML versus LP. All miRNAs with FDR <0.05 are shown. Additional file 4: Table S4. miRNAs that are differentially expressed between the human mammary epithelial subsets: MaSC/basal (MS), luminal progenitor (LP) and mature luminal (ML). Differential expression was assessed using a moderated analysis of variance (ANOVA) F-test for each miRNA between the three populations. The Table shows log2 fold changes for LP versus MS and for ML versus LP. All miRNAs with FDR <0.05 are shown. Additional file 5: Table S5. miRNAs that are differentially expressed in the mouse MaSC/basal (MS) subset compared to the total luminal compartment (LP + ML). Differential expression was assessed using a moderated *t*-test for each miRNA. The Table shows log2 fold changes for MS versus average luminal. All miRNAs with FDR <0.05 are shown. Additional file 6: Table S6. miRNAs that are differentially expressed in the human MaSC/basal (MS) subset compared to the total luminal compartment (LP + ML). Differential expression was assessed using a moderated *t*-test for each miRNA. The Table shows log2 fold changes for MS versus average luminal. All miRNAs with FDR <0.05 are shown. Additional file 7: Table S7. miRNAs that are consistently differentially expressed between MaSC/basal (MS) and the total luminal compartment in both mouse and human. Differential expression was assessed use a moderated *t*-test for each miRNA using both mouse and human data. The Table shows the consensus log2 fold changes for MS versus average luminal across the two species. Additional file 8: Table S8. ROAST tests for inverse correlation between each DE miRNA and its putative mRNA targets in mouse (A) and human (B). The Table shows the number of target genes, the one-sided *P*-value and the FDR for each miRNA. Additional file 9: Table S9. Gene ontology (GO) enrichment analysis of mouse genes regulated by luminal cell-specific miRNAs (A) and MaSC/basal-specific miRNAs (B) in the mouse mammary gland (Ontology groups: *MF* molecular function, *CC* cellular function and *BP* biological process). Additional file 10: Table S10. Gene ontology (GO) enrichment analysis of human genes regulated by luminal cell-specific miRNAs (A) and MaSC/basal-specific miRNAs (B) in the human mammary gland (Ontology groups: *MF* molecular function, *CC* cellular function and *BP* biological process).

## Abbreviations

cDNA: Complementary DNA
Ct: cycle threshold
DE: Differentially expressed
EMT: epithelial mesenchymal transition
ER: Estrogen receptor
FDR: False discovery rate
GO: Gene ontology
HER2: Human epidermal growth factor receptor 2
MaSC: Mammary stem cell
miRNA: microRNA
PR: Progesterone receptor
ROAST: Rotation Gene Set Tests
TCGA: The Cancer Genome Atlas
TSS: Transcriptional start-site
